# Can neoadjuvant chemoradiotherapy before lateral pelvic lymph node dissection improve local control and prognosis in rectal cancer patients with clinically suspected lateral lymph node metastasis? A multicenter lateral node study in China

**DOI:** 10.1186/s12885-024-11867-w

**Published:** 2024-01-23

**Authors:** Zhongshi Xie, Qichen Chen, Bo Feng, Yujuan Jiang, Xin Wang, Wei Xing, Qian Liu, on behalf of the Chinese Lateral Node Collaborative Group

**Affiliations:** 1 Department of Gastrointestinal and Colorectal Surgery, China-Japan Union Hospital of Jilin University, Changchun, China; 2 Department of Colorectal Surgery, State Key Laboratory of Oncology in South China, Guangdong Provincial Clinical Research Center for Cancer,, Sun Yat-sen University Cancer Center, Guangzhou, China; 3 Department of Gastrointestinal Surgery, Ruijin Hospital, Shanghai Jiao Tong University School of Medicine, Shanghai Minimally Invasive Surgery Center, 200025 Shanghai, China; 4 Department of Colorectal Surgery, National Cancer Center/National Clinical Research Center for Cancer/Cancer Hospital, Chinese Academy of Medical Sciences and Peking Union Medical College, 100021 Beijing, China; 5 Department of General Surgery, Peking University First Hospital, 100034 Beijing, China; 6 Department of General Surgery, Hebei Province Hospital of Chinese Medicine, Affiliated Hospital of Hebei University of Chinese Medicine, 050013 Shijiazhuang, Chang’an District China

**Keywords:** Neoadjuvant chemoradiotherapy, Lateral pelvic lymph node dissection, Local control, Prognosis, Safety

## Abstract

**Aims:**

Selective lateral pelvic lymph node (LPN) dissection (LPND) following neoadjuvant chemoradiotherapy (nCRT) for rectal cancer is widely recognized. This study aimed to determine the effects of nCRT before LPND on local control and prognosis of rectal cancer patients.

**Materials and methods:**

Data were retrieved from a prospective database for rectal cancer patients with clinical LPN metastasis receiving total mesorectal excision and LPND at three institutions between January 2012 and December 2019. Selection bias was minimized using propensity score matching (PSM) and short-term and clinical outcomes were compared.

**Results:**

Patients (*n* = 213) were enrolled and grouped as either nCRT (*n* = 97) or non-nCRT (*n* = 116). PSM was used to identify 83 matched pairs. In the matched cohort, nCRT patients had a longer operation duration (310.6 vs. 265.0 min, *P* = 0.001), lower pathological LPN metastasis rate (32.5% vs. 48.2%, *P* = 0.040), and fewer harvested lymph nodes (22 vs. 25, *P* = 0.018) compared to the non-nCRT group. However, after PSM, the two groups had similar estimated overall 3-year survival (79.5% vs. 80.7%, *P* = 0.922), 3-year disease-free survival (66.1% vs. 65.5, *P* = 0.820), and 3-year local recurrence-free survival (88.6% vs. 89.7%, *P* = 0.927). Distant metastasis was the predominant recurrence pattern in the overall (45/58, 77.6%) and matched (33/44, 75.0%) cohorts.

**Conclusions:**

LPND without nCRT is effective and sufficient in preventing local recurrence in patients with LPN metastases. Future prospective randomized controlled studies are warranted to confirm these findings. Since systemic metastasis is the predominant recurrence pattern in patients with LPN metastasis post-LPND, improved perioperative systemic chemotherapy is needed to prevent micrometastasis.

## Introduction

Total mesorectal excision (TME) is a common radical procedure for removing rectal tumors. However, between 10 and 25% of patients with advanced middle-to-lower rectal cancer experience recurrence in the lateral pelvic lymph node (LPN), which is a major site of recurrence following the TME procedure [1, 2]. At present, for the management of LPN metastasis, there is still a dispute between Japan and western countries on the suitability of neoadjuvant chemoradiotherapy (nCRT) as an alternative to LPN dissection (LPND) for patients with clinical LPN metastasis (nCRT + TME vs. TME + LPND). The Japan Society for Cancer of the Colon and Rectum recommends combining TME with LPND to treat stage II and III middle-to-lower rectal cancer [3]. However, the Japanese Clinical Oncology Group (JCOG) determined that the pathologic positive rate of LPN after surgery was only 7%, suggesting that this strategy is not strict enough in case selection and surgical indications, resulting in overtreatment [4]. Meanwhile, a growing volume of literature has demonstrated that nCRT is ineffective in completely eradicating metastatic LPNs without being combined with LPND, and there is a high risk of recurrence with both treatments [5, 6].

LPND and nCRT have gradually converged, and selective LPND following nCRT can eradicate residual lymph nodes while avoiding unnecessary treatment in rectal cancer patients who have reached pathologically complete responses following nCRT [7–9]. However, it is unknown whether local control or patient prognosis can be improved by performing nCRT before LPND. Therefore, we designed a large, multicenter retrospective study and used propensity score-matched (PSM) analysis to evaluate the effects of performing nCRT before LPND on local control and survival.

## Materials and methods

### Patients

Data were retrieved for rectal cancer patients (*n* = 485) with LPN metastasis who received TME and LPND at three institutions from the Chinese Lateral Node Collaborative Group between January 2012 and December 2019 from a prospective database and tumor registry. The Cancer Hospital of the Chinese Academy of Medical Sciences served as the initiator of the study, and the ethics committee of Cancer Hospital, Chinese Academy of Medical Sciences approved this study (NCC 2017-YZ-026, Oct 17, 2017). Patients provided informed consent and all procedures were performed in agreement with the World Medical Association and the Declaration of Helsinki. The study protocol was registered (20/04/2021, NCT04850027) at ClinicalTrials.gov.

**Fig. 1:**
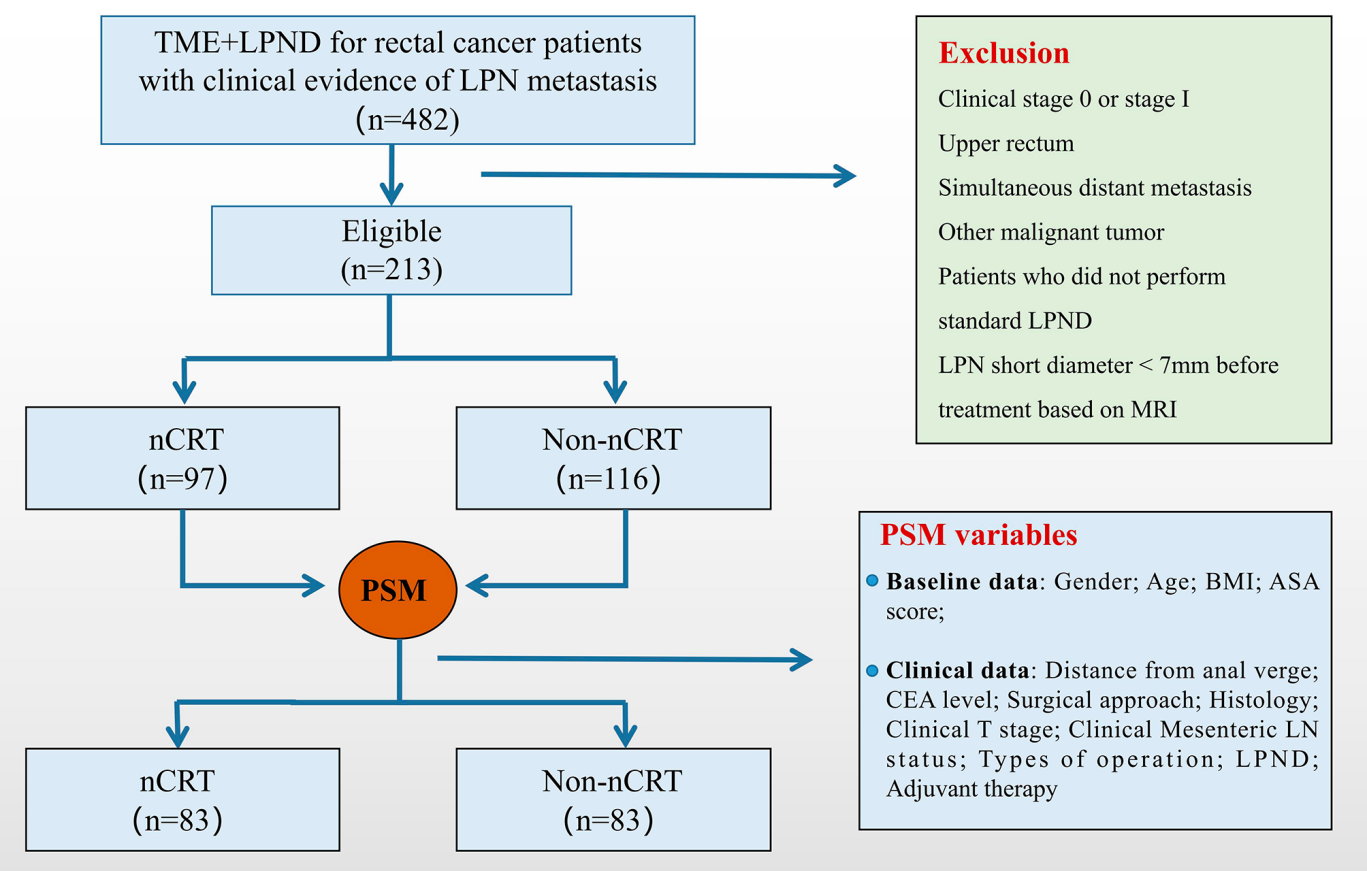
Grouping and PSM flowchart. TME, total mesorectal excision; LPND, lateral pelvic lymph node dissection; nCRT, neoadjuvant chemoradiotherapy; PSM, propensity score matching; LPN, lateral pelvic lymph node; MRI, magnetic resonance imaging; BMI, body mass index; ASA, American Society of Anesthesiologists; LN, lateral lymph node

The inclusion criteria consisted of (1) High-level clinical evidence of LPN metastasis determined by magnetic resonance imaging (MRI); (2) advanced local rectal cancer (cT3-T4/cN+) with the tumor below the peritoneal reflection, and (3) pathology confirmed as adenocarcinoma. Participants were excluded according to the following criteria: (1) concurrent distant metastasis; (2) history of malignant tumor; (3) standard LPND was not performed following the guidelines of the Japanese Society for Cancer of the Colon and Rectum (JSCCR); and (4) complete and standard postoperative adjuvant therapy was not performed. A total of 213 patients were selected and classified as nCRT (*n* = 97) and non-nCRT (*n* = 116) groups depending on the patients’ treatment patterns. Furthermore, PSM was performed to balance variables between groups, resulting in 83 matched pairs (Fig. 1).

### Diagnosis and treatment

Clinical TNM staging and LPN metastases in this study were evaluated and diagnosed by two radiologists using computed tomography (CT) and MRI, where diagnoses met the following three criteria and could be defined as high-level evidence of LPN metastasis: (1) pre-nCRT short diameter of ≥ 0.7 cm; (2) heterogeneous enhancement; and (3) rough edges and irregular shape. The American Joint Committee on Cancer references (8th edition) was used to determine TNM staging in all patients [10]. Events occurring within 30 days following surgery were considered complications and categorized following the Clavien-Dindo system [11].

The patients’ treatment approaches, including the surgical approach (e.g., laparoscopic vs. open) and the decision to perform nCRT, were decided by the patient in consultation with a multidisciplinary team (MDT) comprised of medical and surgical oncologists and radiologists. Among patients with mesorectal fascial (MRF) involvement, multiple lymph node metastases, or a strong desire to preserve the sphincter in ultra-low rectal carcinomas, nCRT was considered and recommended. Furthermore, the treatment strategy for LPN metastases was updated during the study period. Between 2012 and 2017, upfront surgery without nCRT was performed in patients with LPN metastasis After 2018, patients with LPN (≥ 10 mm diameter) underwent nCRT before LPND. These patients received chemotherapy and radiation (capecitabine and 50.4 Gy in 28 fractions) targeting the LPN basin, and TME + LPND was performed 6–8 weeks following nCRT. All patients received LPND after nCRT, regardless of the degree of tumor regression. Adjuvant chemotherapy was administered 4–8 weeks after surgery, including XELOX, mFOLFOX6, or single-agent capecitabine. Patients in the nCRT group underwent adjuvant chemotherapy regardless of the pathological stage, along with six months of perioperative chemotherapy. In the non-nCRT group, high-risk patients in stages II and III (pT4, poor differentiation, tumor perforation, and lymphatic and perineural invasion) received adjuvant chemotherapy after surgery. In addition, patients with pathological LPN metastasis, N2 stage, CRM ≤ 1 mm, and positive margins in the non-nCRT group were recommended to receive chemoradiation (45.0–54.0 Gy in 25–28 fractions, including LPN basin ) in addition to chemotherapy.

### LPND procedure

In this study, the chief surgeons in each institution performed laparoscopic colorectal surgery at least 500 times, and at least 100 cases of LPND were completed. Dissection was performed unilaterally or bilaterally depending on the LPN metastasis location assessed by MRI before treatment. As previously described [9, 12], all patients from the three institutions underwent standard LPND in accordance with JSCCR guidelines, regardless of their response to CRT, and the extent of dissection included four areas: the external iliac, common iliac, internal iliac, and obturator lymph nodes [3]. Caution was taken in identifying the autonomic and obturator nerves and they were completely preserved during dissection.

### Follow-up

During the first three years, patients were examined every three months; after that, they were examined twice a year. Each follow-up entailed a physical examination, assessment of tumor markers, and CT evaluation of the abdomen, chest, and pelvis. A total endoscopy was performed every year. Oncological outcomes were determined by three-year overall survival (OS), recurrence-free survival (RFS), and local RFS (LRFS). Tumor return in the pelvic cavity was considered local recurrence and classified as central (anastomotic, anterior, perineal, or presacral) or lateral, while tumor growth outside the pelvic cavity was considered distant metastasis.

### Statistical analysis

PSM was employed to match patients in the nCRT and non-nCRT groups (caliper = 0.2) to reduce the imbalance. Imbalances were selected based on observations in the original cohort and known potential confounding variables including gender, age, body mass index (BMI), CEA levels, assigned American Society of Anesthesiologists category, surgical approach, types of operation, distance from the anal verge, histology clinical T stage, clinical mesenteric lymph node status, unilateral or bilateral LPND, and adjuvant therapy. These variables were factored into bivariate logistic regression and used to calculate patients’ propensity scores.

Continuous variables were compared by Student’s t-test or Mann–Whitney U test and presented as mean ± standard deviation or as median (range) if assumptions of normal distribution were violated. Categorical variables were compared using the Chi-square test and presented as values (%). The Kaplan–Meier method calculated three-year cumulative OS, RFS, and LRFS. A univariate analysis was performed and assessed using the log-rank test, and when the results were significant, they were subsequently modeled by Cox regression. A *P*-value of < 0.05 was considered significant. Statistical analysis was performed using SPSS v.24.0 (IBM Inc., Armonk, NY, USA).

## Results

### Demographic and clinical data

Table 1 lists the demographic and clinical data before and after PSM. Before PSM, a greater proportion of patients in the nCRT group had ASA grade III (8.3% vs. 1.7%, *P* = 0.027), moderate differentiation (78.4% vs. 65.5%, *P* = 0.039), laparoscopic surgery (91.8% vs. 81.0%, *P* = 0.025), and mesorectal fascial involvement (9.3% vs. 0, *P* = 0.001) than patients in the non-nCRT group. After matching, the variables were not different between groups (*P* > 0.05). Additionally, 65 (78.3%) patients in the nCRT group received adjuvant chemotherapy, 51 (61.4%) patients in the non-nCRT group received adjuvant chemotherapy, and 10 (12.1%) patients received adjuvant chemoradiation in addition to chemotherapy.

**Table 1:** Demographic data and clinical characteristics before and after propensity score matching

Variables	Original cohort			Matched cohort		
	nCRT (*n* = 97)	Non-nCRT (*n* = 116)	*P*	nCRT (*n* = 83)	Non-nCRT (*n* = 83)	*P*
Age (years, mean ± SD)	55.0 ± 11.8	57.6 ± 11.1	0.097	55.6 ± 11.5	57.3 ± 11.2	0.358
Gender			0.650			0.874
Male	59 (60.8)	67 (57.7)		50 (60.2)	49 (59.0)	
Female	38 (39.2)	49 (42.3)		33 (39.8)	34 (41.0)	
BMI (kg/m^2^, mean ± SD)	24.7 ± 3.3	24.1 ± 3.3	0.254	24.6 ± 3.2	24.5 ± 3.3	0.935
ASA category			0.027			1.000
I-II	89 (91.7)	114 (98.3)		81 (97.6)	82 (98.8)	
III	8 (8.3)	2 (1.7)		2 (2.4)	1 (1.2)	
Distance from anal verge, median (range) cm	4 (1–8)	4 (1–8)	0.113	4 (1–8)	4 (1–8)	0.486
CEA level (ng/ml)			0.654			0.751
≥5	41 (42.3)	51 (44.0)		32 (38.6)	34 (41.0)	
<5	46 (57.7)	65 (56.0)		51(61.4)	49 (59.0)	
LPN short-diameter before nCRT (cm, mean ± SD)	1.3 ± 0.6	1.1 ± 0.5	0.103	1.2 ± 0.5	1.2 ± 0.6	0.930
Surgical approach			0.025			0.787
Open	8 (8.2)	22 (19.0)		7 (8.4)	8 (9.6)	
Laparoscopic	89 (91.8)	94 (81.0)		76 (91.6)	75 (90.4)	
Histology			0.039			0.222
Moderate	76 (78.4)	76 (65.5)		64 (77.1)	57 (68.7)	
Poor/Mucinous/signet	21 (21.6)	40 (34.5)		19 (22.9)	26 (31.3)	
Clinical T stage			0.107			0.755
T_1_ -T_2_	5 (5.2)	8 (6.9)		5 (6.0)	6 (7.2)	
T_3_-T_4_	92 (94.8)	108 (93.1)		78 (94.0)	77 (92.8)	
Clinical Mesenteric LN status			0.191			0.360
Positive	85 (87.6)	94 (81.0)		66 (79.5)	61 (73.5)	
Negative	12 (12.4)	22 (19.0)		17 (20.5)	22 (26.5)	
(y) Clinical T stage			-			-
T_1_-T_2_	26 (26.8)	-		25 (30.1)	-	
T_3_-T_4_	71 (73.2)	-		58 (69.9)	-	
(y) Clinical Mesenteric LN status			-			-
Positive	54 (55.7)	-		50 (60.2)	-	
Negative	43 (44.3)	-	-	33 (39.8)	-	
LPN short-diameter after nCRT (cm, mean ± SD)	0.7 ± 0.4	-		0.7 ± 0.3	-	-
Type of operation			0.792			0.759
LAR	44 (45.3)	60 (51.7)		38 (45.8)	40 (48.2)	
APR	44 (45.3)	48 (41.4)		37 (44.6)	38 (45.8)	
Hartmann procedure	6 (6.2)	5 (4.3)		5 (6.0)	4 (4.8)	
TPE	3 (3.1)	3 (2.6)		3 (3.6)	1 (1.2)	
LPND			0.473			0.310
Bilateral	32 (33.0)	33 (28.4)		28 (33.7)	22 (26.5)	
Unilateral	65 (67.0)	83 (71.6)		55 (66.3)	61 (73.5)	
Adjuvant therapy	79 (81.4)	85 (73.3)	0.158	65 (78.3)	61 (73.5)	0.468
Chemotherapy	79 (81.4)	73 (63.0)		65 (78.3)	51 (61.4)	
Chemoradiation + chemotherapy	0 (0)	12 (10.3)		0 (0)	10 (12.1)	
Mesorectal fascial involvement			0.001			0.245
Yes	9 (9.3)	0 (0)		2 (2.4)	0 (0)	
No	88 (90.7)	116 (100.0)		81 (97.6)	83 (100.0)	

Note: nCRT, neoadjuvant chemoradiotherapy; BMI, body mass index; ASA, American Society of Anesthesiologists; LN, lymph node; LAR, low anterior resection; APR, abdominal perineal resection; TPE, total pelvic exenteration; LPND, lateral pelvic lymph node dissection; SD, standard deviation

### Short-term outcomes

Surgical outcomes and complications are presented in Table 2. There was a longer operative time for the nCRT group than the non-CRT group after PSM (310.6 vs. 265.0 min, *P* = 0.001), but estimated blood loss during surgery did not differ (50 vs. 50 ml, *P* = 0.281). The groups did not have differences in terms of grade 1–5 (21.7% vs. 14.5%, *P* = 0.226) and grade 3–5 (9.6% vs. 8.4%, *P* = 0.787) postoperative complications. After matching, the two groups of patients had similar postoperative hospital stays (11.6 vs. 10.9 d, *P* = 0.626), and no perioperative deaths were reported.

**Table 2:** Surgical outcomes and pathological results before and after propensity score matching

Variables	Original cohort			Matched cohort		
	nCRT (*n* = 97)	Non-nCRT (*n* = 116)	*P*	nCRT (*n* = 83)	Non-nCRT (*n* = 83)	*P*
Operative time (min, mean ± SD)	306.1 ± 90.9	281.3 ± 102.2	0.048	310.6 ± 95.8	265.0 ± 79.5	0.001
Estimated blood loss, median (range) ml	50 (10 − 1,700)	50 (10 − 2,500)	0.329	50 (10 − 1,700)	50 (10 − 1,100)	0.281
Postoperative complications (Grade 1–5)	20 (20.6)	18 (15.5)	0.333	18 (21.7)	12 (14.5)	0.226
Postoperative complications (Grade 3–5)	9 (9.3)	10 (8.6)	0.867	8 (9.6)	7 (8.4)	0.787
Mortality	0 (0)	1 (0.9)	1.000	0 (0)	0 (0)	1.000
Postoperative hospital stay (days, mean ± SD)	11.2 ± 9.5	12.9 ± 10.0	0.208	11.6 ± 10.2	10.9 ± 7.4	0.626
(y) Pathological T stage			< 0.001			< 0.001
T_0_ -T_2_	31 (31.9)	14 (12.1)		28 (33.7)	7 (8.4)	
T_3_-T_4_	66 (68.1)	102 (87.9)		55 (66.3)	76 (91.6)	
(y) Pathological mesenteric LN status			0.001			0.003
Positive	45 (46.4)	81 (69.8)		38 (45.8)	57 (68.7)	
Negative	52 (53.6)	35 (30.2)		45 (54.2)	26 (31.3)	
PCR rate	10 (10.3)	-	-	9 (10.8)	-	-
Down T staging rate	64 (65.9)	-	-	58 (69.9)	-	-
Down N staging rate	70 (72.2)	-	-	63 (75.9)	-	-
Pathological LPN metastasis	30 (30.9)	57 (49.1)	0.007	27 (32.5)	40 (48.2)	0.040
Total LNs harvested, median (range)	22 (6–62)	26 (7–77)	0.042	22 (6–62)	25 (8–77)	0.018
LPNs harvested, median (range)	8 (1–16)	8 (1–17)	0.652	8 (1–16)	7 (1–15)	0.244
Circumferential Resection Margin			0.593			1.000
Positive	2 (2.1)	1 (0.9		1 (1.2)	0 (0)	
Negative	95 (97.9)	115 (99.1)		82 (98.8)	83 (100.0)	

Note: nCRT, neoadjuvant chemoradiotherapy; SD, standard deviation

### Pathological results

Pathological results pre- and post-PSM are presented in Table 2. In the matched cohort, the rates of pathological complete response (PCR), down T staging, and down N staging after nCRT, were 10.8%, 69.9%, and 75.9%, respectively. There was a lower rate of pathological LPN metastasis in the nCRT group compared with the non-nCRT group (32.5% vs. 48.2%, *P* = 0.040). nCRT significantly reduces the amount of harvested lymph nodes (22 vs. 25, *P* = 0.018). No difference in the positive circumferential resection margin (1.2% vs. 0, *P* = 1.000) was observed between groups.

### Survival analysis

**Fig. 2:**
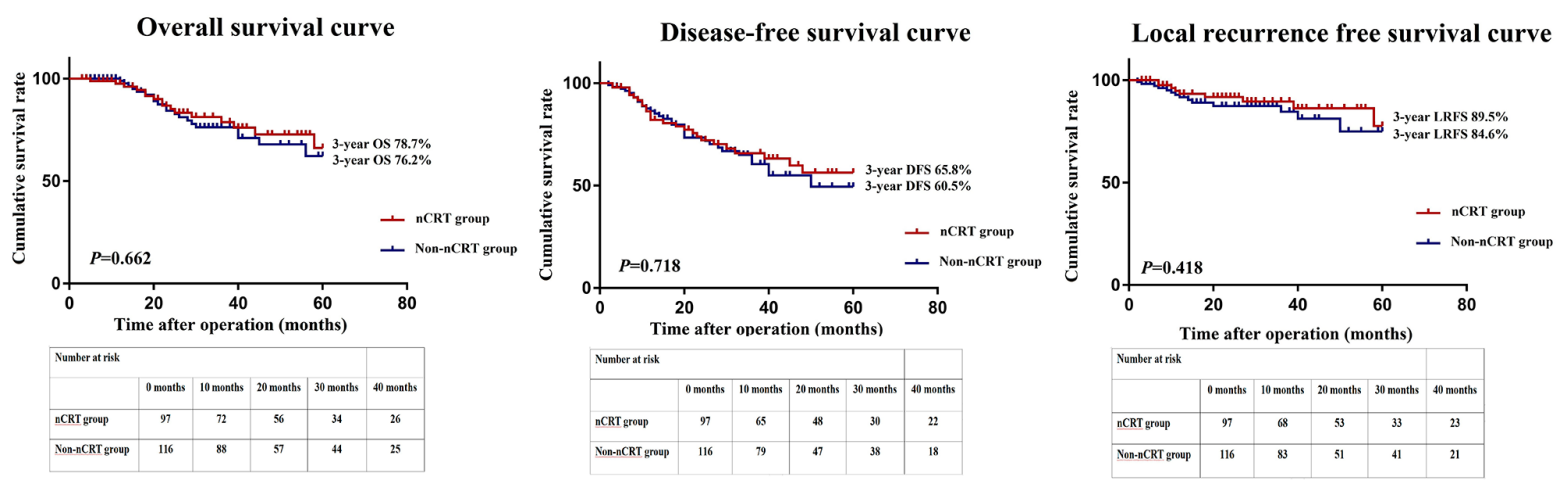
OS curve, RFS curve, and LRFS curve for patients in the nCRT and non-nCRT groups before PSM. OS, overall survival; RFS, recurrence-free survival; LRFS, local recurrence-free survival; nCRT, neoadjuvant chemoradiotherapy; PSM, propensity score matching

**Fig. 3:**
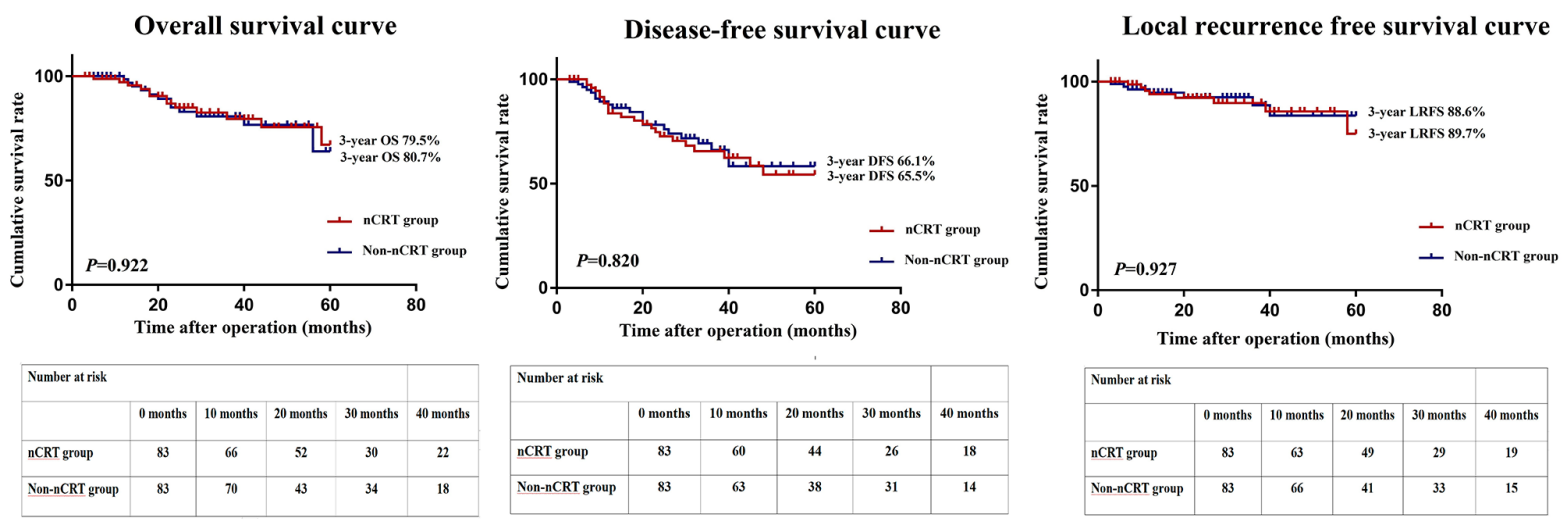
OS curve, RFS curve, and LRFS curve for patients in the nCRT and non-nCRT groups after PSM. OS, overall survival; RFS, recurrence-free survival; LRFS, local recurrence-free survival; nCRT, neoadjuvant chemoradiotherapy; PSM, propensity score matching

The mean follow-up periods for the nCRT versus non-nCRT groups were 38.8 and 37.0 months, respectively, in the overall cohort, and 38.1 and 36.2 months, correspondingly, in the matched cohort. Before PSM, the two groups did not differ in the estimated three-year OS (78.7% vs. 76.2%, *P* = 0.662), three-year RFS (65.8% vs. 60.5, *P* = 0.718), and three-year LRFS (89.5% vs. 84.6%, *P* = 0.418) **(**Fig. 2). Similarly, there were no differences in the estimated three-year OS (79.5% vs. 80.7%, *P* = 0.922), three-year RFS (66.1% vs. 65.5, *P* = 0.820), and three-year LRFS (88.6% vs. 89.7%, *P* = 0.927) between the two groups after PSM (Fig. 3).

In addition, Table 3 shows recurrence patterns up to three years after surgery in both groups before and after PSM. Before PSM, the proportion of local recurrences (9.3% vs.12.1%, *P* = 0.513) and distant recurrences (19.6% vs. 22.4%, *P* = 0.615) was comparable between the nCRT and non-nCRT groups. After PSM, both groups had a similar proportion of local recurrences (9.6% vs.8.4%, *P* = 0.787) and distant recurrences (20.5% vs. 19.3%, *P* = 0.846). It is worth noting that whether in the overall cohort (45/58, 77.6%) or the matched cohort (33/44, 75.0%), the predominant recurrence was distant metastasis.

**Table 3:** Recurrence pattern up to three years after surgery before and after propensity score matching

Variables	Original cohort			Matched cohort		
	nCRT (*n* = 97)	Non-nCRT (*n* = 116)	*P*	nCRT (*n* = 83)	Non-nCRT (*n* = 83)	*P*
Overall recurrence	26 (26.8)	32 (27.6)	0.898	23 (27.7)	21 (25.3)	0.725
Local recurrence	9 (9.3)	14 (12.1)	0.513	8 (9.6)	7 (8.4)	0.787
Central pelvic	6 (6.2)	10 (8.6)		5 (6.0)	6 (7.2)	
Lateral pelvic	4 (4.1)	6 (5.2)		3 (3.6)	3 (3.6)	
Distant metastasis	19 (19.6)	26 (22.4)	0.615	17 (20.5)	16 (19.3)	0.846
Lung	12 (12.4)	15 (12.9)		10 (12.0)	11 (13.3)	
Liver	10 (10.3)	11 (9.5)		9 (10.8)	8 (9.6)	
Bone	6 (6.2)	8 (6.9)		5 (6.0)	5 (6.0)	
Peritoneum	1 (1.0)	3 (2.6)		1 (1.2)	2 (2.4)	
Lymph node	1 (1.0)	2 (1.7)		1 (1.2)	2 (2.4)	

Note: nCRT, neoadjuvant chemoradiotherapy

Prognostic factors influencing RFS and LRFS in the overall cohort identified by univariate and multivariate analyses are shown in Table 4. For the univariate analyses, histology, operative type, lymphatic invasion, pathological mesorectal LN metastasis, pathological LPN metastasis, and grade 3–4 postoperative complication influenced RFS and LRFS (*P* < 0.05). The pathological T stage also influenced LRFS (*P* < 0.05). For the multivariate analyses, pathological LPN metastasis (HR: 3.11; 95% CI: 1.68–5.77, *P* < 0.001) and pathological mesorectal LN metastasis (HR: 4.16; 95% CI: 1.76–9.87, *P* = 0.001) were revealed as independent prognostic factors for RFS and LRFS, respectively.

**Table 4:** Univariate and multivariate analyses for DFS and LRFS in the overall cohort

Variables	RFS				LRFS			
	Univariate analysis		Multivariate analysis		Univariate analysis		Multivariate analysis	
	HR (95%CI)	*P*	HR (95%CI)	*P*	HR (95%CI)	*P*	HR (95%CI)	*P*
Gender: male/female	1.11 (0.65–1.91)	0.704			0.93 (0.47–1.85)	0.843		
Age at operation	0.99 (0.96–1.01)	0.258			0.98 (0.95–1.01)	0.270		
ASA score: I-II/III	2.53 (0.35–18.28)	0.359			1.45 (0.20-10.64)	0.713		
Pre-nCRT CEA level (≥ 5/<5 ng/ml)	1.12 (0.65–1.91)	0.682			1.19 (0.60–2.36)	0.622		
Neoadjuvant chemoradiotherapy (yes/no)	0.86 (0.51–1.48)	0.718	1.30 (0.73–2.33)	0.370	0.81 (0.41–1.62)	0.662	1.24 (0.61–2.54)	0.552
Histology ( Poor, Mucinous or signet/moderate)	2.72 (1.29–5.77)	0.009	1.24 (0.57–2.72)	0.587	3.57 (1.26–10.09)	0.017	1.17 (0.42–3.25)	0.761
Operative type: laparoscopic/open	0.37 (0.21–0.66)	0.001	0.56 (0.29–1.09)	0.088	0.36 (0.18–0.73)	0.004	0.65 (0.29–1.46)	0.292
Lymphatic invasion (yes/no)	5.12 (2.64–9.20)	< 0.001	1.33 (0.57–3.10)	0.508	4.91 (1.61–14.93)	0.005	2.38 (0.88–6.44)	0.089
Perineural invasion (yes/no)	1.24 (0.65–2.36)	0.516			2.44 (0.95–7.42)	0.088		
Pathological T stage (T_3_-T_4_/T_1_-T_2_)	1.84 (0.90–3.77)	0.094			2.89 (1.02–8.24)	0.047	1.77 (0.59–5.32)	0.311
Pathological mesorectal LN status (positive/negative)	3.00 (1.51–5.96)	0.002	1.70 (0.79–3.65)	0.174	5.93 (2.67–13.18)	< 0.001	4.16 (1.76–9.87)	0.001
Pathological LPN metastasis (yes/no)	4.00 (2.28–7.02)	< 0.001	3.11 (1.68–5.77)	< 0.001	4.56 (1.60-12.98)	0.004	2.00 (0.65–6.17)	0.228
Postoperative complication (yes/no)	1.39 (0.43–4.47)	0.578			2.70 (0.82–8.91)	0.103		
Grade 3–4 Postoperative complication (no/yes)	1.84 (1.01–3.38)	0.049	1.71 (0.92–3.20)	0.093	2.25 (1.07–4.73)	0.033	2.12 (0.87–4.61)	0.058
Adjuvant therapy (yes/no)	1.36 (0.52–4.89)	0.542			2.31 (0.45–9.32)	0.635		

Note: RFS, recurrence-free survival; LRFS, local recurrence-free survival; HR, hazard ratio; 95%CI, 95% confidence interval; ASA, American Society of Anesthesiologists, nCRT, neoadjuvant chemoradiotherapy; CEA,; LN, lymph node; LPN, lateral pelvic lymph node

## Discussion

As stated previously, for the management of LPN metastasis, there is no consensus between Japanese and Western countries, and nCRT and LPND are mutually exclusive treatments. In recent years, the treatment strategy of selective LPND after nCRT has been recognized and supported, and the indications of selective LPND after nCRT have been gradually established [7–9, 13]. In the present study, 7 mm was used as the threshold value for suspected LPN metastasis, and performing nCRT before LPND did not significantly improve local control or long-term survival.

There is controversy over LPND because it is technically difficult to perform and the pelvic sidewall is anatomically complex. nCRT may not only cause tissue edema and fibrosis, which makes LPND even more difficult to perform but also potentially inhibits the immune system and increases the risk of infection at the incision site and in the lung and pelvis [14]. Our study found no differences between the two groups in intraoperative bleeding, surgical complications, or postoperative length of hospital stay, except for the significantly longer duration of surgery in the nCRT group (matched cohort: 310.6 vs. 265.0 min, *P* = 0.001). In a study by Ogura et al. assessing 107 patients that received TME with LPND after nCRT, there was a median operation length of 461 min and median blood loss of 115 ml. In agreement with our results, there were no differences in surgical complications [15]. This supports our conclusion that nCRT followed by LPND is safe and feasible in experienced and well-equipped high-volume centers, even if it increases surgical duration, as it does not translate into higher morbidity.

The amount of lymph nodes dissected is important to consider when evaluating the quality of surgery and determining prognosis [16]. However, nCRT could reduce the number of lymph nodes dissected, which may be related to the depletion of lymphocytes by local radiotherapy and the replacement of fibrous connective tissue [17]. The present study demonstrated that although fewer lymph nodes were dissected in the nCRT group than in the non-nCRT group (matched cohort: 22 vs. 25, *P* = 0.018), there was still a median of greater than 12 lymph nodes dissected. In addition, the number of LPN harvested indirectly reflects the quality of LPND, which also affects the evaluation of tumor stage and adjuvant therapy. In this study, the median number of LPN harvested between the two groups was similar before (8 vs. 8, *P* = 0.652) and after (8 vs. 7, *P* = 0.244) matching, which also indicated that nCRT did not affect the quality of LPND and pathological evaluation.

nCRT resulted in satisfactory therapeutic effects, with a PCR rate of 10.8%, a down T staging rate of 69.9%, and a down N staging rate of 75.8% in the matched cohort, consistent with those reported previously [18, 19]. Selective LPND following nCRT could eradicate residual lymph nodes in rectal cancer patients who attained pathologically complete responses following nCRT while avoiding unnecessary treatment [7–9]. In a previous study, we assessed the indications for performing LPND following nCRT, and patients with an LPN short diameter of < 7 mm following nCRT and good pathological histology did not require LPND after nCRT, thus avoiding excessive surgical trauma [9]. This study spanned more than 8 years, and treatment strategies were constantly updated and revised. All patients included in this study had an LPN diameter of ≥ 7 mm before nCRT and underwent LPND regardless of the short diameter after nCRT. The pathological LPN metastasis rates were 32.5% for the nCRT patients and 48.2% for the non-nCRT patients in the matched cohort. We hypothesized that for nearly 16% of patients with clinical LPN metastases, LPND could be avoided through nCRT assuming that the established indications for LPND after nCRT were followed.

Our study demonstrated no significant improvements in three-year OS (79.5% vs. 80.7%, *P* = 0.922), three-year RFS (66.1% vs. 65.5, *P* = 0.820), and three-year LRFS (88.6% vs. 89.7%, *P* = 0.927) between the nCRT and non-nCRT groups. We investigated the postoperative recurrence patterns and the primary cause of treatment failure as distant metastasis [overall cohort: 77.6% (45/58); matched cohort: 75% (33/44)], suggesting that patients with LPN metastasis were in the advanced stages and tended to develop systemic metastasis. In recent years, the concept of a multidisciplinary comprehensive treatment strategy has positively improved the prognosis for patients with LPNM. Since nCRT is less effective in reducing the risk of systemic metastasis for rectal cancer patients, adjuvant chemotherapy is needed to eliminate potential micro-metastases [20]. However, the effectiveness of adjuvant therapy is limited by poor compliance and high complication risk. In this study, nCRT patients are supposed to receive adjuvant chemotherapy regardless of the pathological stage according to established guidelines. However, nearly 20% of these patients did not receive adjuvant chemotherapy for various reasons. Therefore, the management and administration of adjuvant chemotherapy is a prominent issue. We suggested that it may be considered under the premise of controlling toxicity to supplement strengthening chemotherapy before, during, and after radiotherapy (totally neoadjuvant therapy), or even replacing radiotherapy with intensive chemotherapy, with the goal of minimizing micro-metastasis, decrease the rate of distant metastasis, which would optimize the treatment strategy of LPN metastasis [21, 22]. Objective data and research are needed to verify and support the above conclusions in the future.

Our study has several limitations that need consideration. First, we did not analyze urinary and sexual dysfunction, the major postoperative complications related to LPND. Therefore, the safety of LPND after nCRT cannot be completely measured and reflected. Second, this study was retrospective, participants were not randomized, and we could not preclude the possibility of selection bias. A small proportion of patients in the non-nCRT group received adjuvant chemoradiation in addition to chemotherapy, which could interfere with the analysis of the results between the two groups. Furthermore, as clinical LPN metastasis might be over-staged based on preoperative MRIs, the demographic characteristics and clinical staging were different between the CRT and non-CRT groups. However, the same diagnostic criteria for LPN metastasis were used for both groups, while performing PSM to reduce selection bias; data before and after PSM were retained, reflecting the outcomes of nCRT for LPN metastasis in China. Third, because this study spanned 8 years, only 45.5% (97/213) of patients with clinical LPNM underwent nCRT, and this figure may not be consistent with that for the conventional treatment strategies. A prospective, randomized study will help to further confirm the oncological effect of performing nCRT before LPND in patients with LPN metastasis.

## Conclusion

In summary, LPND without nCRT is effective and sufficient in preventing local recurrence for patients with LPN metastases, and randomized controlled trials are further warranted. Since systemic metastasis is the predominant recurrence in patients with LPN metastasis following LPND, improved perioperative systemic chemotherapy is needed to prevent micro-metastasis.

## Data Availability

The datasets generated and/or analysed during the current study are not publicly available due to the data is confidential patient data but are available from the corresponding author on reasonable request.
